# Minoxidil may suppress androgen receptor-related functions

**DOI:** 10.18632/oncotarget.1886

**Published:** 2014-04-08

**Authors:** Cheng-Lung Hsu, Jai-Shin Liu, An-Chi Lin, Chih-Hsun Yang, Wen-Hung Chung, Wen-Guey Wu

**Affiliations:** ^1^ Division of Hematology-Oncology, Departments of Internal Medicine, Chang Gung Memorial Hospital, Chang Gung University, Taoyuan 333, Taiwan, ROC; ^2^ Department of Dermatology, Chang Gung Memorial Hospital, Chang Gung University, Taoyuan 333, Taiwan, ROC; ^3^ Institute of Bioinformatics and Structural Biology, National Tsing Hua University, Hsinchu 300, Taiwan, ROC

**Keywords:** minoxidil, androgen, androgen receptor, androgenetic alopecia, prostate cancer

## Abstract

Although minoxidil has been used for more than two decades to treat androgenetic alopecia (AGA), an androgen-androgen receptor (AR) pathway-dominant disease, its precise mechanism of action remains elusive. We hypothesized that minoxidil may influence the AR or its downstream signaling. These tests revealed that minoxidil suppressed AR-related functions, decreasing AR transcriptional activity in reporter assays, reducing expression of AR targets at the protein level, and suppressing AR-positive LNCaP cell growth. Dissecting the underlying mechanisms, we found that minoxidil interfered with AR-peptide, AR-coregulator, and AR N/C-terminal interactions, as well as AR protein stability. Furthermore, a crystallographic analysis using the AR ligand-binding domain (LBD) revealed direct binding of minoxidil to the AR in a minoxidil-AR-LBD co-crystal model, and surface plasmon resonance assays demonstrated that minoxidil directly bound the AR with a *K_d_* value of 2.6 μM. Minoxidil also suppressed AR-responsive reporter activity and decreased AR protein stability in human hair dermal papilla cells. The current findings provide evidence that minoxidil could be used to treat both cancer and age-related disease, and open a new avenue for applications of minoxidil in treating androgen-AR pathway-related diseases.

## INTRODUCTION

The androgen-androgen receptor (AR) pathway is involved in a wide range of physiological development and disease processes. Notable in this latter context is androgenetic alopecia (AGA). In AGA, the androgen-AR pathway may affect hair growth in a paradoxical pattern at puberty, inhibiting hair growth in the scalp of genetically predisposed individuals but enhancing hair growth at most other body sites outside the scalp area. AGA is the most frequent cause of hair loss in men [1]. Balding may be inherited in a polygenetic manner, and genetic predispositions, including AR polymorphisms in chromosome Xq12 [2] and single-nucleotide polymorphisms (SNPs) in chromosome 20p11 [3, 4] and chromosome 3q26 [5], have been linked to AGA.

The AR is a transcription factor that belongs to the steroid hormone receptor superfamily. Prior to androgen binding, the AR is associated with the chaperone proteins HSP70 and HSP90. Upon androgen binding, the androgen-AR complex undergoes a conformational change and translocates to nucleus, where it forms a dimer on target gene promoters and turns on target gene transcription. This process is assisted by an array of coregulators [6].

Testosterone, the major androgen in the circulation, is converted to the more potent androgen, dihydrotestosterone (DHT), via 5α-reductase in tissue. Two types of 5α-reductase isoenzymes have been described, type I and type II, the latter of which is more highly expressed in AGA follicles than in follicles of normal controls [7]. The AR is also expressed at significantly higher levels in balding than non-balding scalp follicles in AGA [8]. Men with Kennedy's disease, who harbor dysfunctional ARs with long polyQ repeats in the N-terminus, have significantly thicker hair and a lower risk of AGA [9]. Furthermore, the AR coregulator Hic5/ARA55 is more richly expressed in balding frontal scalp papilla cells than in occipital area cells [10]. There is also a literature report that DHT-inducible DKK1 (Dickkopf-related protein 1) from balding dermal papilla causes apoptosis in follicular cells [11]. These data support the concept that the activity of the androgen-AR signaling pathway may play an important role in AGA pathogenesis.

Minoxidil was introduced clinically in the early 1970s as an anti-hypertension medication, but was found to have the side effect of hypertrichosis, an abnormal amount of hair growth over the body [12]. Currently, minoxidil is approved for topical therapy of AGA and is available as 2% and 5% solutions and as a 5% foam formulation. Both 2% and 5% solutions were shown to arrest hair lost on the vertex and frontal scalp in most patients, but exhibited lower efficacy in stimulating hair regrowth [1]. In minoxidil responders, maintenance therapy was needed. The therapeutic mechanism of minoxidil action in AGA is uncertain, but a number of possibilities, including vasodilation [13], angiogenesis [14], enhanced cell proliferation [15], modulation of potassium channel conductance [16] and regulation of prostaglandin [17, 18], have been proposed.

Previous studies have consistently concluded that minoxidil does not act directly through an androgen effect [19, 20]. However, it nevertheless influences the androgen-AR pathway-dominant disease, AGA, prompting us to hypothesize that minoxidil does indeed affect AR-related functions. To test this speculative relationship between minoxidil and AR, we performed a series of experimental and modeling studies.

## RESULTS

### Minoxidil suppresses AR transactivation and target gene expression

**Figure 1:**
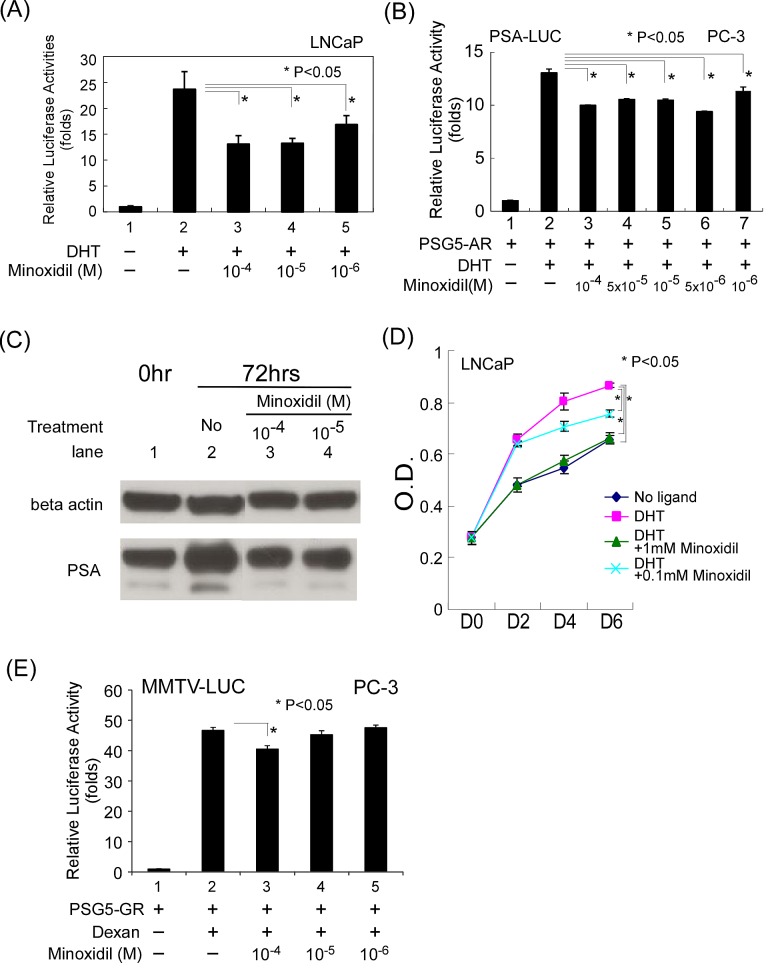
Minoxidil suppresses AR-related functions (A-B) AR transcriptional activity in reporter assays. Cells in 24-well plates were transfected as indicated below, and after incubating for 16 h, were treated with ethanol, 10 nM DHT, or 1–100 μM minoxidil for an additional 16 h. Thereafter, luciferase activity in cell lysates was determined and normalized to protein concentration. Relative luciferase activity was calculated as described in Materials and methods. (A) LNCaP cells were transfected with 1,000 ng of MMTV-Luc reporter plasmid. (B) PC-3 cells were transfected with 500 ng of pSG5-AR and 500 ng of PSA-LUC reporter plasmid. (C) PSA protein expression. LNCaP cells seeded on 100-mm dishes the previous day were treated with vehicle (DMSO), 10 nM DHT, or 10–100 μM minoxidil. After 72 h, total cell lysates were prepared and analyzed for AR and PSA expression by Western blotting as described in Materials and methods. (D) LNCaP cell growth. LNCaP cells were plated at 5 × 104 cell/well in 24-well plates and incubated with or without 1 nM DHT and 100–1000 μM minoxidil for 2 to 6 d. Cell growth was measured by MTT assay as described in Materials and methods. (E) GR transcription activity. The same procedure was performed as in (B), except the plasmids pSG5-GR and MMTV-LUC and the hormone dexamethasone (Dexan; 1 μM) were used.

Minoxidil has been used to treat the AR-related disease, AGA, for more than two decades, suggesting that it may interfere with AR-related functions. To investigate this possibility, we first tested minoxidil in an AR transcription reporter assay using LNCaP prostate cancer cells, which harbor an endogenous AR with a T877A mutation. Cells were transiently transfected with an AR-responsive MMTV (mouse mammalian tumor virus)-Luc construct and treated with different concentrations of minoxidil. Minoxidil concentrations were chosen based on most common reports in the literature: 0.1 to 10 μM for oral intake and about 1 mM for topical application of 5% minoxidil (100 mM) in skin tissue (Regaine 5% minoxidil topical solution; monograph, February 2013), assuming 1.7% absorption. In *in vitro* organ culture or animal studies, high concentrations, ranging from 1-100 mM, have been reported in skin tissue [31, 32]. After treating with different concentrations of minoxidil (1 to 100 μM), cells were harvested and cellular extracts were assayed for luciferase activity. As shown in Fig. 1A, minoxidil suppressed AR reporter activity at the concentrations tested.

To further clarify whether minoxidil suppresses human AR target gene transcription, we co-transfected PC-3 cells with PSG5-AR and a PSA-Luc reporter construct containing 1.5 kb of the prostate-specific antigen (PSA) promoter linked to the luciferase gene. As shown in Fig. 1B, minoxidil suppressed AR transcriptional activity in this PSA-Luc reporter assay. Furthermore, as shown in Fig. 1C (PSA, lanes 3 and 4 vs. lane 2), this phenomenon translated into a decrease in PSA protein level after treatment with minoxidil for 72 h. These data further support the conclusion that minoxidil is capable of suppressing AR-related functions.

Clinically, anti-androgens are used to treat prostate cancer. To further test minoxidil suppression of AR-related function, we tested the effects of minoxidil on the growth of LNCaP cells, an androgen-sensitive prostate cancer cell line that, as noted above, endogenously expresses AR. As shown in Fig. 1D, minoxidil suppressed DHT-dependent LNCaP cell growth in concentration-dependent manner. Notably, at the highest concentration, cell growth was suppressed to a level comparable to that observed in the absence of DHT. These data provide the first demonstration that minoxidil can suppress AR-related functions, including AR transcription and AR-related cell growth.

To assess possible nonspecific effects of minoxidil at high concentrations, we performed control studies, testing different concentrations of minoxidil (1-100 μM) on glucocorticoid receptor (GR) transcriptional activity. As shown in Fig. 1E, the highest concentration minoxidil (100 μM) did affect GR transcriptional activity in reporter assays in PC-3 cells, whereas lower concentrations (1-10 μM) did not. These data suggest that minoxidil, a small hydrophobic molecule, may have multiple targets in the cell when used at high concentrations.

### Minoxidil blocks FxxLF motif-containing peptide-AR, cofactor-AR, and AR N-C interactions

**Figure 2:**
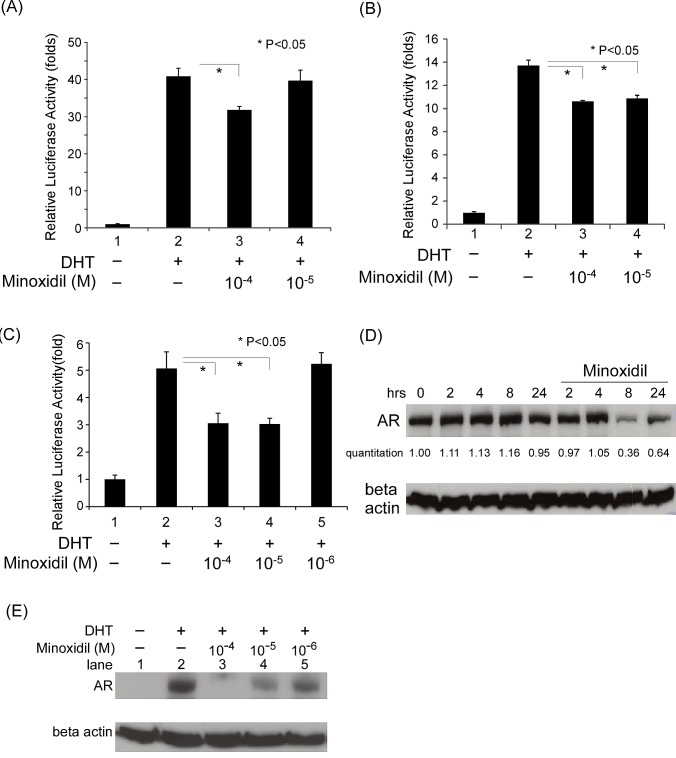
The mechanism of minoxidil suppression of AR-related functions (A-C) Transcriptional activity in reporter assays. Cells in 24-well plates were transfected as indicated below, and after incubating for 16 h, were treated with ethanol, 10 nM DHT, or 1–100 μM minoxidil for an additional 16 h. Thereafter, luciferase activity in cell lysates was determined and normalized to protein concentration. Relative luciferase activity was calculated as described in Materials and methods. (A) PC-3 cells were transfected with 350 ng GAL4-DBD-3-18, 350 ng VP16-AR, and 300 ng pG5-Luc plasmids. (B) PC-3 cells were transfected with 350 ng GAL4-DBD-ARA54C, 350 ng VP16-AR, and 300 ng pG5-Luc plasmids. (C) PC-3 cells were co-transfected with 350 ng of pCDNA3-flag-hAR-N (amino acids 1–506), 350 ng pCDNA3-hAR-C (amino acids 556–919), and 300 ng MMTV-Luc plasmids. (D-E) AR stability. LNCaP cells were seeded in dishes and incubated for 24 h. Thereafter, 10 μg/ml cyclohexamide and 10 nM DHT were added along with 100 μM of minoxidil for pulse-chase time-course studies (D) or different concentrations of minoxidil (E) followed by an additional 24-h incubation. AR and β-actin (loading control) protein levels were determined by Western blotting.

We next investigated the mechanism underlying the ability of minoxidil to suppress AR-related functions. Full transcriptional activity of the AR requires the assistance of coregulators. Phage display and real AR coregulator analyses have shown that an FxxLF-like motif in coregulators mediates coregulator-AR interactions. To determine whether disruption of these interactions contributes to the suppression of AR-related function by minoxidil, we first examined the effect of minoxidil on the interaction between an FxxLF-containing peptide and the AR. In these experiments, PC-3 cells were co-transfected with an FxxLF-containing peptide (GAL4-DBD-3-18), an expression plasmid for full-length AR (VP16-AR), and a pG5-Luc construct. After treating with DHT and different concentrations of minoxidil, cell extracts were assayed for luciferase activity. As shown in Fig. 2A, minoxidil inhibited FxxLF-containing peptide-AR interaction-dependent reporter activity in a concentration-dependent manner, suggesting that minoxidil interferes with peptide-AR interactions. Using this same experimental paradigm, we further tested minoxidil effects on coregulator-AR interactions, substituting ARA54C, a C-terminal construct of the coactivator ARA54 (androgen receptor associated protein 54), for the FxxLF-containing peptide. As was the case for the isolated FxxLF-containing peptide, the transcription-enhancing effect of ARA54C was inhibited by minoxidil, consistent with the idea that minoxidil interferes with the interaction of the ARA54 C-terminus with the AR (Fig. 2B).

Formation of an AR dimer, which is required for transcriptional activity, involves an interaction between AR N- and C-termini mediated by the AR N-terminal FxxLF motif. Co-expressing truncated AR N- and C-termini recapitulated this AR N-C interaction, turning on target gene expression (Fig. 2C, lanes 1 and 2). As shown in Fig. 2C, minoxidil at concentrations ranging from 10 to 100 μM suppressed this induction of transcriptional activity, suggesting that minoxidil also disrupts AR N- and C-terminal interactions, providing an addition mechanism for minoxidil suppression of AR-related function.

### Minoxidil decreases AR stability

AR blockade has been shown to influence AR protein stability [33]. To test minoxidil effects on AR protein stability, we treated cells with 10 μM minoxidil for different lengths of time in the presence of cyclohexamide to block new protein synthesis and examined the time course of AR protein disappearance by Western blotting. As shown in Fig. 2D, minoxidil induced a decrease in AR protein that was most evident 8 h after treatment, at which point AR protein levels were 36% of pretreatment values. We further found that this decrease in AR protein levels was dependent on minoxidil concentration, as shown in Fig. 2E. These observations suggest that the mechanism of action of minoxidil may include a decreased in AR stability.

### DHT-AR-LBD and minoxidil co-crystal model

**Figure 3:**
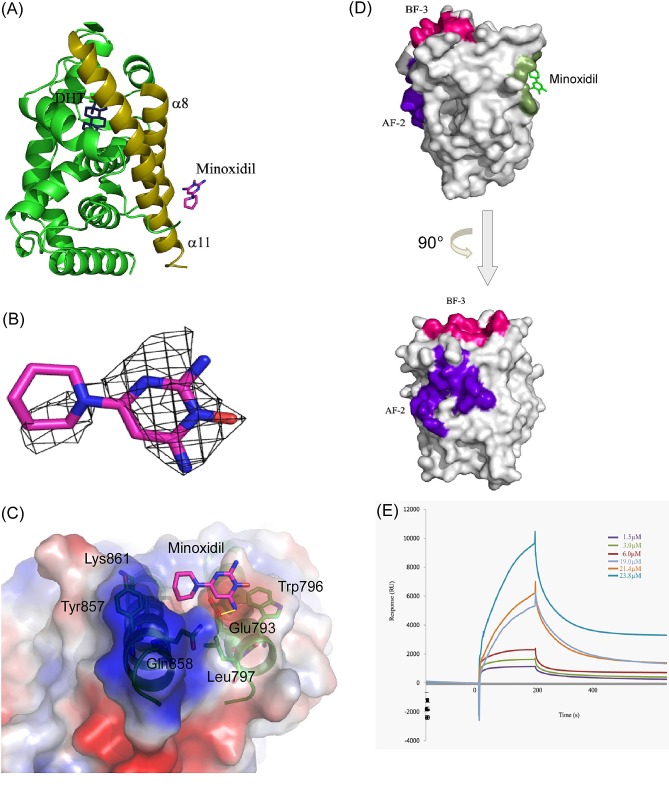
Minoxidil-AR-LBD complex structure (A) Overall structure of the AR-LBD. The AR-LBD is illustrated as a green ribbon model. DHT lies in the pocket, whereas the minoxidil molecule is located in the groove formed by α8 and α11 helices (dark yellow). DHT is depicted as sticks with carbon and oxygen atoms in blue and red, respectively. Minoxidil is shown as sticks with carbon, oxygen, and nitrogen atoms in magenta, red, and blue, respectively. (B) The 2*Fo-Fc* electron density map for minoxidil contoured at the 1.0σ level is illustrated as a grey mesh. Minoxidil (magenta) is shown in stick representation. (C) The minoxidil-binding groove. Minoxidil, depicted as sticks with carbon, oxygen, and nitrogen atoms in magenta, red, and blue, respectively, lies in the groove. Residues involved in minoxidil binding are shown in green stick representations. The α8 and α11 helices of the AR-LBD are illustrated as a green ribbon model. Hydrogen bond contact is illustrated by a yellow line. (D) Location of the different binding sites on AR-LBD. *Upper figure:* Surface representation of the binding sites of minoxidil (green), AF-2 (purple), and BF-3 (red). Minoxidil is drawn as a stick representation. *Lower figure:* Same as in upper figure, but rotated counterclockwise 90° along the Y-axis to show the AF-2 site. (E) Interactions between minoxidil and AR-LBD protein measured by SPR. Representative SPR sensorgrams for AR-LBD (1.5–25 μM) binding to minoxidil. Minoxidil was immobilized onto the surface of the sensor chip CM5, and the AR-LBD protein was injected at concentrations of 1.5, 3.0, 6.0, 19.0, 21.4, and 23.8 μM.

The ability of minoxidil to reduce AR protein stability, taken together with the apparent interference with cofactor-AR and AR N- and C-terminal interactions by minoxidil, suggests that minoxidil may bind directly to the AR. To confirm this hypothesis, we attempted to co-crystalize a mixture of minoxidil, AR-LBD, and DHT. AR-LBD was successfully crystalized in the presence of DHT and minoxidil, and the structure of the resulting complex was diffracted to 2.4 Å resolution (Fig. 3A). An electron density map of a single subunit could be built from residues 670 to 918 in the protein, revealing an asymmetric unit with orthorhombic symmetry. Finally, the model was refined to yield R and free-R values of 17.0% and 19.9%, respectively. The overall crystallographic statistics are given in Supplemental Table 1.

DHT was found to be conjugated in a pocket surrounded by four residues, Asn705, Gln711, Arg752 and Thr877, with which it formed hydrogen bonds. Interestingly, the minoxidil molecule could be modeled as an additional electron density map that was localized to a previously unreported groove formed by α8 and α11 helices (Fig. 3A, 3B). Several mobile residues from these two helices, including Glu793, Trp796, Leu797, Tyr857, Gln858 and Lys861, were found near the minoxidil molecule (<5 Å). Minoxidil contains N and O atoms that may act as functional groups to form hydrogen bonds with these nearby residues. One such interaction involves the amine group in the pyrimidine ring of minoxidil, which is predicted to form a hydrogen bond with the hydroxyl group of Glu793 (3.1 Å). Other residues provide a hydrophobic environment that stabilizes the compound (Fig. 3C). Although this position is somewhat removed from the AR-LBD AF2 (activation function 2) site, which is the main cofactor binding site, it is possible that minoxidil interferes with coregulator interactions in the context of full-length AR (Fig. 3D).

We further performed isothermal titration calorimetry experiments to analyze the binding affinity between the drug and protein. After having obtained clear evidence that the drug bound to the protein, we proceeded to determine the binding affinity by SPR, analyzing the interaction of different concentrations of AR-LBD protein with minoxidil, measured as relative units (RU), as a function of time (s). As shown in Fig. 3E, the binding affinity between the minoxidil and AR-LBD protein, expressed as *K_d_*, was calculated to be 2.6 μM. These results are consistent with our resolved structure and results of functional assays.

### Minoxidil suppresses AR transactivation in skin cells

**Figure 4:**
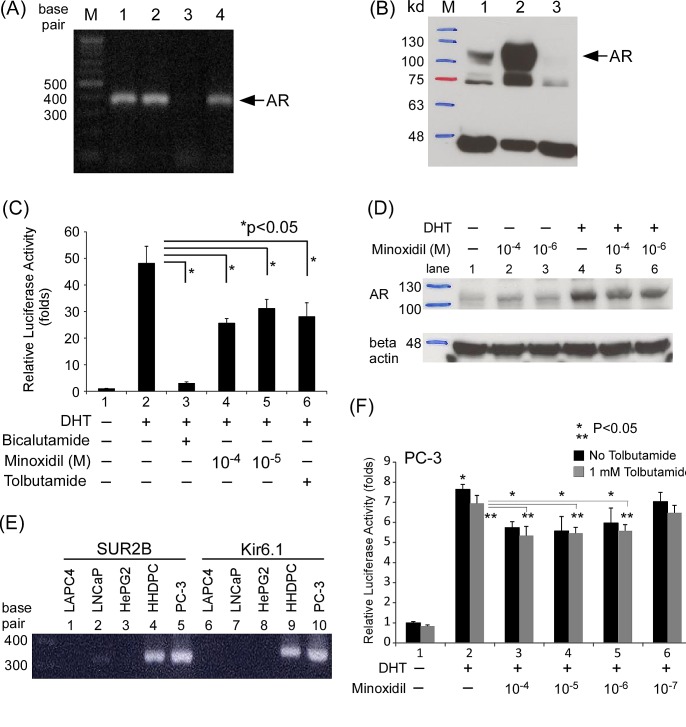
Minoxidil suppresses AR-related functions in skin cells (A-B) AR expression in cells and cell lines at the (A) mRNA and (B) protein levels. Lane 1, HHDPCs; lane 2, LNCaP cells; lane 3, PC-3 cells. (C) HHDPCs in 24-well plates were transfected with 1,000 ng of MMTV-Luc reporter plasmid. After 16 h, ethanol, 10 nM DHT, 10–100 μM minoxidil, and/or 1 mM tolbutamide was added and cells were incubated for an additional 16 h. Luciferase activity in cell lysates was normalized to protein concentration. Relative luciferase activity was calculated as described in Materials and methods. (D) After seeding HHDPCs in dishes and culturing for 24 h, 10 μg/ml cyclohexamide, 10 nM DHT, and different concentrations of minoxidil were added and cells were incubated for an additional 2 h. AR and β-actin (loading control) protein levels were determined by Western blotting. (E) Potassium channel subunit expression in different cells and cell lines. Prostate cancer cell lines: LAPC4, LNCaP, and PC-3; liver cancer cell line: HePG2; skin cells: HHDPCs. PC-3 cells expressed the same potassium channel subtype (SUR2B/Kir6.1) as HHDPCs. (F) Tolbutamide did not reverse minoxidil effects in AR transactivation reporter assays in PC-3 cell. The same procedure as in (C) was performed except with the addition of different concentrations of tolbutamide

All of the above data were obtained in prostate cancer cell lines or in *in vitro* studies. To extend these findings to skin cells, we tested whether minoxidil has similar effects in HHDPCs (human hair dermal papilla cells) using an AR reporter assay. AR expression in HHDPCs was confirmed at mRNA (Fig. 4A) and protein (Fig. 4B) levels. As shown in Fig. 4C, minoxidil suppressed AR transcriptional activity in a concentration-dependent manner (compare lanes 4 and 5 to lane 2). We further tested minoxidil effects on AR protein stability in HHDPCs. As shown in Fig. 4D, minoxidil induced a concentration-dependent reduction in AR protein stability. These data provide further evidence that the efficacy of minoxidil in treating AGA may involve suppression of AR-related functions.

Minoxidil has been proposed to act as a potassium channel opener in the context of AGA treatment, an action that is primarily associated with the SUR2B/Kir6.1 potassium channel subtype in hair follicles [31, 34]. Tolbutamide, a potassium channel blocker that has been reported to antagonize minoxidil effects on hair growth [31], suppressed AR transcriptional activity in a reporter assay in HHDPCs, as shown in Fig. 4C (compare lane 6 to lane 2). Tests of HHDPCs and various cancer cell lines further showed that HHDPCs expressed the same subtype of potassium channel as that found in the prostate cancer cell line PC-3, which lacks an endogenous AR (Fig. 4E). Notably, minoxidil suppressed AR transcriptional activity in PC-3 cells, but this effect was not blocked by tolbutamide (Fig. 4F), suggesting that the suppressive effect of minoxidil on AR transcriptional activity is not a potassium channel-related action.

## DISCUSSION

For more than 20 years, minoxidil has been used to treat AGA, which is an androgen-AR pathway-dominant disease. The scientific literature suggests that minoxidil acts mainly on the hair cycle, shortening telogen [35] and increasing hair diameter [36]. Minoxidil actions as an anti-hypertension agent have been mainly attributed to its potassium channel-opening effect, which has been linked to the hypertrichosis phenomenon associated with minoxidil [37]. However, this mechanism is not compatible with certain findings, including the observations that potassium channel antagonists are unable to block minoxidil effects and potassium channels are not expressed in hair follicle cells [38, 39]. Although a specific subtype of potassium channel has been identified in human hair follicles [31, 34], our data suggest that minoxidil modulation of AR activity does not involve this pathway.

AGA is closely associated with androgen-AR pathway activity. Previous studies using a golden Syrian hamster model found no anti-androgenic potential of minoxidil on androgen-dependent cutaneous structures [19]. However, female animals were used in these studies, and testosterone, which can be converted to estradiol in hair follicles [40], rather than DHT was chosen. Furthermore, minoxidil treatment increases 17β-hydroxysteroid dehydrogenase and 5α-reductase activity, which may influence testosterone metabolism [41]. Recently, the prostaglandin D2 receptor G protein-coupled receptor 44 pathway was shown to play an important role in AGA pathogenesis [17]. Minoxidil treatment increases prostaglandin E2 levels, but whether it influences prostaglandin D2-related pathways is unknown.

Our characterization of the relationship between minoxidil and AR showed that minoxidil interferes with AR-related functions, decreasing AR transcriptional activity, reducing expression of targets at the protein level, and suppressing AR-positive LNCaP cell growth. Furthermore, our mechanistic studies showed that the suppressive effect of minoxidil on AR-related functions reflected its ability to directly bind to the AR; interfere with AR-peptide, AR-coregulator and AR N-C interactions; and reduce AR protein stability. It has been proposed that the AR and its downstream pathways, but not androgen, may be alternative targets in androgen-AR pathway-dominant diseases. Specifically, ASC-J9, a curcumin derivative that is in clinical trial for acne treatment, has been reported to possess AR-degradation–enhancing activity [33, 42].

The potassium channel family, which includes the K_ATP_ channel, is a diverse group of membrane proteins that has been linked to cell proliferation [43]. These channels can be expressed in normal prostate gland tissue and prostate cancer [44]. However, potassium channel density may be inversely correlated with the metastatic potential of prostate cancer, with higher expression in LNCaP cells (low metastatic ability) and lower expression in PC-3 cells (high metastatic rate) [45]. Minoxidil has been shown to enhance the growth of PC-3 cells which express K_ATP_ channel but lack an endogenous AR in serum-free conditions, and some potassium channel blockers suppress the growth of prostate cancer cell lines [44]. Our data showed that not all prostate cancer cell lines express the same subtype of potassium channel (Fig. 4E) and we further found that minoxidil suppressed the growth of AR-expressing LNCaP cells in a concentration-dependent manner. A number of common drugs that are used chronically for other indications, such as rapamycin, metformin, β-blockers, angiotensin-blockers and aspirin, can protect against cancer [46, 47]. Apart from its actions as a K_ATP_ channel opener in the treatment of hypertension and AGA, minoxidil may directly bind to the AR and suppress AR-related functions, including androgen-sensitive LNCaP cell growth. These data may suggest the potential of minoxidil in the treatment of prostate cancer.

Interestingly, our structural studies demonstrated that minoxidil is chelated at a novel, previously unreported groove in the AR. The residues surrounding the groove provide hydrophobicity at the surface, whereas the inner core exhibits hydrophilic properties, implying selectivity for binding factors. Moreover, the groove is also located opposite to and at a distance from AF-2 and BF-3 sites [48]; accordingly, it may represent a new candidate site for the interaction of AR coregulators (Fig. 3D). To date, there have been few reports on AR structure and none has described the role of the groove identified here in AR action. Mutation of a nearby residue, Cys784, to Tyr784 in the α8 helix was reported to cause the loss of transactivation activity in a female patient [49]. In our model, variations in this region resulted in steric clashes or changes in secondary structure. By occupying this grove, minoxidil may hinder the physical association of interacting proteins, thereby disrupting downstream regulation of AR transactivation. Taken together with mutagenesis data, our structural findings suggest that this groove may be important for binding of AR-interacting proteins during transactivation. The minoxidil-AR-LBD co-crystal model thus may provide a platform for the further development of drugs for the treatment of AGA and other androgen-AR pathway-related diseases.

## MATERIALS AND METHODS

### Materials and plasmids

DHT, bicalutamide, cyclohexamide, tolbutamide, and minoxidil were obtained from Sigma Chemical Co. (St. Louis, MO). The cell lines PC-3, LNCaP, LAPC4, and HepG2 were purchased from American Type Culture Collection (Manassas, VA). Human hair dermal papillae cells (HHDPCs) were purchased from ScienCell Research Laboratories (San Diego, CA). The plasmids pSG5-AR, pSG5-GR, Gal-4-ARA54C, Gal-4-peptide, pCDNA3-AR-N, pCDNA3-AR-C, pCMX-VP16-AR, MMTV-Luc, PSA-Luc, and pG5-Luc were constructed as described previously [21, 22]. Anti-AR (N-20) and anti-PSA (C-19) antibodies were purchased from Santa Cruz Biotechnology, Inc. (Santa Cruz, CA). The anti-β-actin antibody (MAB1501) was obtained from Millipore (Billerica, MA).

### AR-LBD protein purification and minoxidil-AR-LBD co-crystallization

cDNA for the human AR ligand-binding domain (AR-LBD; amino acids 663-919) was amplified from pSG5-AR by polymerase chain reaction (PCR) and inserted into the pET28c vector (Novagen, San Diego, CA). Protein expression and purification were as described previously [23]. The DHT-AR-LBD complex was crystallized at room temperature using the hanging-drop vapor-diffusion method. Crystal Screen 2 (Hampton Research, Aliso Viejo, CA) solution number 20 and modified buffers were used to monitor DHT-AR-LBD crystal formation. After crystals had reached 0.7 × 0.7 × 2.5 mm with a rhombus shape, the DHT-AR-LBD crystals were transferred to a solution containing 10 mM minoxidil for 30 min at room temperature. The complex crystal was flash-cooled by dipping in a cryoprotectant solution consisting of the reservoir solution containing 20% (v/v) glycerol, and then was placed in a cold stream at 100K.

The 2.4 Å dataset was collected at the BL13B at the National Synchrotron Radiation Research Center in Hsinchu, Taiwan, using an ADSC Quantum 315r CCD detector. Indexing and processing of the measured intensity data were performed using the HKL2000 software package [24]. The crystal belonged to the space group *P*2_1_2_1_2_1_, with unit cell parameters of *a* = 55.43 Å, *b* = 65.54 Å, *c* = 70.38 Å, and α = β = γ = 90°. The structures were determined by the molecular replacement method AMoRe, using the unliganded AR-LBD structure (PDB code: 1T7T) as the search model [25]. Refinement was carried out using the REFMAC5 program [26] and coupled to ARP/wARP [27]. The refined model was checked using the PROCHECK program [28]. Data collection and refinement statistics are summarized in Supplemental Table 1. The coordinates have been deposited in the Protein Data Bank under the entry 4K7A.

### Transient transfection reporter gene assay

The PC-3 human prostate cancer cell line was grown in Dulbecco's minimal essential medium containing 10% fetal bovine serum, 2 mM L-glutamine, and 10 U/ml penicillin; the LNCaP cell line and HHDPCs were maintained in RPMI-1640 medium containing 10% fetal bovine serum and antibiotics. Culture media for all cell lines were phenol red-free, and charcoal-treated fetal bovine serum was used in DHT-related assay. For transfection, cells were plated in 24-well plates and transfected with plasmids using a Superfect kit (Qiagen, Valencia, CA), as described by the manufacturer. After 16 h of incubation, the cells were treated with ethanol (vehicle control), steroid hormones, or different concentrations of minoxidil for an additional 16 h and then harvested for luciferase assays. Luciferase activity in whole-cell extracts was measured using a luciferase assay kit, as described by the manufacturer (Promega, Madison, WI) and normalized to the protein concentration in the respective extract. The results were presented as means ± standard deviations (SD) of triplicate determinations, expressed relative to untreated control cells (defined as 1); three independent transfections were performed.

### Cell growth assay

LNCaP cells were plated at 5 × 10^4^ cell/well in 24-well plates and incubated with or without 1 nM DHT and different concentrations of minoxidil for 2 to 6 d. Cell growth was measured by MTT [3-(4,5-dimethythiazol-2-yl)-2,5-diphenyl tetrazolium bromide] assay as described previously [29]. Briefly, at each time point, 50 μl of a 1 mg/ml MTT solution was added to each well containing 500 μl medium, and plates were incubated at 37°C for 3 h. After adding 500 μl of isopropyl alcohol to dissolve the converted dye, the absorbance of each well was measured at 570 and 650 nm using a DU 640B spectrophotometer (Beckman, Fullerton, CA) according to the manufacturer's protocol. Data in figures are means ± SD of at least triplicate wells.

### Western blot analysis

Western blot analyses were performed as previously described in detail [29] using anti-AR (1:250), anti-PSA (1:500), and anti-β-actin (1:40,000) primary antibodies.

### Protein stability and degradation

The effect of minoxidil on AR protein stability was assessed by first seeding and incubating LNCaP cells for 24 h. The culture medium was then replaced with medium containing 10 μg/ml cyclohexamide alone or with 10 nM DHT or different concentrations of minoxidil, and plates were incubated for different lengths of time. Thereafter, cell lysates were analyzed for AR and β-actin by Western blotting.

### Surface plasmon resonance (SPR) assay

SPR experiments were carried out on a Biacore 3000 instrument (GE Healthcare). All experiments were performed at 25°C as described previously [30]. For binding analysis, minoxidil was immobilized on a CM5 SPR biosensor chip (SA chip; GE Healthcare) at a flow rate of 5 μl/min. Different concentrations of AR-LBD protein were injected over the minoxidil-coated surfaces at a flow rate of 40 l/min. Surfaces were regenerated by injection of 0.02% (w/v) sodium dodecyl sulfate (SDS) and 1.0 M NaCl. Following subtraction of data from the control flow cell to eliminate non-specific binding, all curves were fitted by the numerical integration method using BIA evaluation 3.2 software, as described in the BIA evaluation handbook (GE Healthcare).

### RT-PCR

RT-PCR was used to investigate the expression of mRNA for the ATP-sensitive potassium (K_ATP_) channel subunits, Kir6.1 and SUR2B, in HHDPCs and cancer cell lines. mRNA was converted to cDNA using the avian myeloblastosis virus (AMV) reverse transcription (RT) system (Promega, Madison, WI), as described by the manufacturer. PCR amplification was performed under standard conditions using Taq DNA polymerase (Invitrogen, Carlsbad, CA) and the following primer pairs: AR (M20132.1), 5'-CAG GAA TTC CTG TGC ATG AAA GCA CTG C-3' (forward) and 5'-TCA CTG GGT GTG GAA ATA GAT GGG C-3' (reverse); SUR2B (NM_005691), 5'-GCT GAA GAA TAT GGT CAA ATC TC-3' (forward) and 5'-TGG AGT GTC ATA TTC TAA AAT A-3' (reverse); Kir6.1 (NM_004982), 5'-CAT CTT TAC CAT GTC CTT CC-3' (forward) and 5'-GTG AGC CTG AGC TGT TTT CA-3' (reverse). pSG5-AR served as a positive control in AR studies, and HHDPCs served as a positive control in K_ATP_ channel studies. The target bands were sequenced and compared with published sequences [31].

### Statistical analysis

Data are expressed as means ± SEM. Differences between two groups were assessed using an unpaired two-tailed Student's *t-*test.

## SUPPLEMENTARY TABLE


