# Supplementary material for: Minoxidil may suppress androgen receptor-related functions
Source: Oncotarget. 2014 Apr 8;5(8):2187–97. doi: 10.18632/oncotarget.1886 (PMC4039155; doi:10.18632/oncotarget.1886)
Supplement: Supplementary file 1 [file oncotarget-05-2187-s001.pdf]

Minoxidil may suppress androgen receptor-related functions

Supplemental Table 1. Minoxidil-DHT-AR-LBD

Summary of Structures and Crystallographic Statistics

Data collection	
X-ray wavelength (Å)	0.97622
Space group	$P2_12_12_1$
Unit cell (Å)	*a* = 55.43 Å, *b* = 65.54 Å, *c* = 70.38 Å,
Resolution (Å)	30.0–2.4
Unique reflections	10075
Completeness (%)[a]	96.1 (86.2)
I/σ (Outer shell)	21.38 (3.3)
Average Redundancy	10.3
R_{sym} (%)[a,b]	0.107
No. of protein molecules per a.u.	1
**Refinement**	
R value (%)[c]	19.2
R_{free} value (%)[d]	26.8
R.M.S.D. bond length (Å)	0.012
R.M.S.D. bond angle (°)	1.674
Average B factor ($Å^2$)	30.9
PDB accession code	4K7A

[a]Numbers in parenthesis denote values for the highest resolution shell.

[b]$R_{sym} = \Sigma / I - <I> // \Sigma (I)$.

[c]$R = \Sigma /Fo - Fc// \Sigma (Fo)$, where *Fo* and *Fc* are observed and calculated structure factors, respectively.

[d]R_{free} was calculated similarly with a randomly selected set of reflections consisting of 5% of total reflections that were excluded from refinement.
